# Prevalence of Lower Urinary Tract Symptoms in Pregnant Women in Yazd, Iran: A Cross-Sectional Study

**DOI:** 10.7759/cureus.84190

**Published:** 2025-05-15

**Authors:** Parisa Ghadiri Harati, Seyed Majid Hosseini, Atiyeh Javaheri, Farideh Dehghan Manshadi, Alireza Akbarzadeh Baghban

**Affiliations:** 1 Department of Physiotherapy, Faculty of Rehabilitation, Shahid Beheshti University of Medical Sciences, Tehran, IRN; 2 Department of Gynecology, Faculty of Medicine, Shahid Sadoughi University of Medical Sciences, Yazd, IRN; 3 Department of Biostatistics, Proteomics Research Center, School of Allied Medical Sciences, Shahid Beheshti University of Medical Sciences, Tehran, IRN

**Keywords:** diabetes, lower urinary tract symptoms, pregnant women, prevalence, quality of life

## Abstract

Introduction: Lower urinary tract symptoms (LUTS) are a common and significant concern during pregnancy; however, research on LUTS in pregnant women in Iran, particularly in Yazd, is limited. This study aimed to investigate the prevalence and associated factors of LUTS in pregnant women in Yazd, Iran.

Methods: A cross-sectional study was conducted among pregnant women attending routine antenatal visits in Yazd between October 2022 and May 2023. Demographic and clinical data were obtained from patients’ medical records. LUTS were assessed using the structured Persian version of the International Consultation on Incontinence Questionnaire for Female LUTS (ICIQ-FLUTS) long form.

Results: Out of the 422 pregnant women initially enrolled, 397 met the inclusion criteria. The most prevalent bladder filling symptom was nocturia (83.9%), followed by increased frequency (52.1%) and urgency (46.9%). Voiding symptoms were less common, with interrupted stream (27.2%) and hesitancy (11.3%) being the most reported. Stress urinary incontinence was the most prevalent type of incontinence (45.1%). Multivariate logistic regression analysis showed that advancing gestational age was significantly associated with increased odds of both bladder filling (odds ratio (OR) = 1.09; p < 0.001) and urinary incontinence symptoms (OR = 1.02; p = 0.044). While higher body mass index (BMI) was associated with increased odds of urinary incontinence, the association was not statistically significant (p = 0.078). No significant predictors were found for voiding symptoms.

Conclusion: This study highlights the high prevalence of LUTS, especially nocturia and stress urinary incontinence, among pregnant women in Yazd. Advancing gestational age and higher BMI were associated with increased symptoms, while gestational diabetes was linked to greater voiding difficulties. These findings emphasize the importance of routine screening and early intervention to minimize the impact of LUTS on maternal quality of life.

## Introduction

Lower urinary tract symptoms (LUTS) are a common and significant health concern during pregnancy, affecting a large proportion of pregnant women worldwide [1]. Recent studies show that the prevalence of LUTS in pregnant women ranges from 81% to 99% [2,3]. While urinary incontinence has long been the primary focus of LUTS research, it is important to recognize that LUTS encompasses a broader spectrum of symptoms, including storage, voiding, and post-micturition symptoms. These symptoms can cause considerable discomfort, reduce quality of life (QOL), and may persist long after childbirth, with the potential to worsen over time [4]. The anatomical and physiological changes during pregnancy, such as hormonal fluctuations, increased pressure on the bladder from the growing uterus, and alterations in pelvic floor muscle function, are believed to contribute to the onset and severity of these symptoms [5].

The prevalence of LUTS is known to increase with advancing gestational age. Other contributing factors include maternal age, obesity, constipation, and parity, among others [6-10]. Despite the negative impact of LUTS on women’s QOL [11], many affected individuals do not seek medical care, often due to a lack of awareness or social stigma. Therefore, improving awareness of commonly underreported symptoms such as urinary incontinence is crucial. Preventive strategies, such as lifestyle modifications and early interventions, may help maintain lower urinary tract function during pregnancy.

To date, no published studies have specifically examined the prevalence of LUTS in the general population of pregnant women in Yazd, Iran. Moreover, there is a general lack of data on LUTS in pregnant Iranian women. This study, therefore, aimed to investigate the prevalence, associated risk factors, and QOL impact of LUTS among pregnant women in Yazd, providing valuable regional insights into this understudied topic.

## Materials and methods

Study design and participants

This descriptive cross-sectional study evaluated the prevalence of LUTS among 397 healthy pregnant women attending routine antenatal visits at outpatient clinics. Participants were enrolled consecutively from 10 October 2022 to 30 May 2023. The sample size was calculated as 397 based on a 95% confidence level, a 5% margin of error, and an expected prevalence derived from previous studies. The inclusion criteria were pregnant women aged between 16 and 46 who attended clinics. Exclusion criteria included pregnant women with known pre-existing urinary tract disorders (e.g., genetic or neurological causes), current urinary tract infection (UTI), urolithiasis, use of medications affecting bladder function, chronic neurologic disease, or psychiatric illness, as well as multiple pregnancies or conditions associated with increased uterine volume, such as polyhydramnios, to minimize potential confounding effects on LUTS [12]. All exclusions were made strictly based on the predefined inclusion and exclusion criteria, ensuring consistency and minimizing any selection bias. This approach allowed for a more accurate assessment of LUTS in the targeted population. Participants who met the inclusion criteria and were not excluded were considered for final analysis.

Data collection instruments

Data were collected using a structured questionnaire in Persian, which consisted of two main sections, detailed as follows.

Demographic and Medical Data

Age, body mass index (BMI), parity, history of diabetes, and current gestational age were obtained.

International Consultation on Incontinence Questionnaire for Female Lower Urinary Tract Symptoms

The Persian version of International Consultation on Incontinence Questionnaire for Female Lower Urinary Tract Symptoms (ICIQ-FLUTS) was utilized to screen participants for LUTS. The ICIQ-FLUTS questionnaire is a well-established and widely used tool for assessing LUTS, enabling precise identification of the severity and type of LUTS in patients. This questionnaire was selected for its high reliability, ease of use, and ability to evaluate various aspects of LUTS [13,14].

The questionnaire consists of 13 items on LUTS experienced in the four weeks before the survey, with responses given on a 5-point scale (0-4) based on the presence and severity of symptoms. The symptoms assessed were related to the storage phase, voiding phase, and incontinence. Participants' level of discomfort caused by these symptoms was assessed using an 11-point scale (0-10), which was subsequently grouped into four categories: 0 = not bothered, 1-3 = mildly bothered, 4-7 = moderately bothered, and 8-10 = severely bothered.

Ethics approval and consent to participate

This study was conducted in accordance with the principles of the Declaration of Helsinki. The study protocol, including access to and use of clinical data, was approved by the Research Ethics Committee of Shahid Beheshti University of Medical Sciences (approval ID: IR.SBMU.RETECH.REC.1400.611; date: November 28, 2021). All participants provided verbal informed consent for their involvement in the study, and they were informed about the study's objectives and their potential contribution to UI research before giving consent. Additionally, women who were unable to comprehend the questionnaire were excluded from the study. For this study, the latest International Continence Society (ICS) definitions and terminology for LUTS were adopted [15].

Statistical analysis

Data analysis was performed using IBM SPSS Statistics for Windows, Version 26.0 (Released 2019; IBM Corp., Armonk, New York, United States). Descriptive statistics included means with standard deviations (SD) and medians with interquartile ranges (IQR). A significance threshold of p < 0.05 was considered statistically significant. The chi-square test was used to compare the prevalence of urinary symptoms and QOL between women with and without diabetes. Multivariate logistic regression analysis was used to identify factors associated with bladder filling symptoms, voiding symptoms, and urinary incontinence. The results of these analyses are presented as adjusted odds ratio (aOR) with 95% confidence interval (95% CI).

## Results

A total of 422 pregnant women were initially enrolled; however, 25 participants were excluded based on the inclusion criteria. Thus, the final analysis included 397 pregnant women. Demographic and clinical characteristics are summarized in Table 1. The mean maternal age was 28.77 ± 5.70 years, the mean BMI was 28.06 ± 4.89 kg/m², and the mean gestational age was 25.44 ± 10.13 weeks.

**Table 1:** Demographic and clinical characteristics of pregnant women. SD: standard deviation; BMI: body mass index; DM: diabetes mellitus; GDM: gestational diabetes mellitus.

Parameters	Mean ± SD or n (%)
Age (years)	28.77 ± 5.70
Weight (kg)	72.82 ± 13.00
Height (cm)	161.16 ± 6.71
BMI (kg/m^2^)	28.06 ± 4.89
Gestational age (weeks)	24.55 ± 10.13
Gravidity count	
1	140 (35.3)
2	141 (35.5)
3	65 (16.4)
4	35 (8.8)
≥5	16 (4.0)
Diabetes	
Normal	297 (74.8)
Pre-pregnancy DM	36 (9.1)
GDM	64 (16.1)

Participants' ages ranged from 16 to 42 years, with an average age of 28.77 years, and the majority were multiparous. Among the participants, 9.1% had pre-pregnancy diabetes and 16.1% were diagnosed with gestational diabetes mellitus (GDM), accounting for 100 women in total.

The results of the ICIQ-FLUTS questionnaire are presented in Table 2. Reported bladder filling symptoms included increased frequency, nocturia, and urgency. Among these, nocturia, defined as waking to void one or more times per night, was the most prevalent, reported by 83.87% of participants, and was associated with a negative impact on QOL in 36.8% of cases. Increased urinary frequency was reported by 52.1% and urgency by 46.9%, with 35% and 26.4% of affected women, respectively, reporting an adverse impact on QOL. Among these three symptoms, increased frequency was the most commonly reported by women with GDM; however, this difference was not statistically significant.

Voiding symptoms reported by participants included bladder pain, hesitancy, straining, and interrupted stream. Among these, the interrupted stream had the highest prevalence at 27.2%, followed by bladder pain (31.7%), hesitancy (11.33%), and straining (4.28%). In terms of urinary incontinence, stress urinary incontinence (SUI) was the most commonly reported type, with a prevalence of 45.1%, followed by mixed urinary incontinence at 41.3%. The distribution and frequencies of incontinence types are detailed in Table 2. 

**Table 2:** Occurrence of LUTS in pregnant women. QOL: quality of life; LUTS: lower urinary tract symptoms.

Symptoms	Prevalence, n (%)	QOL score, n (%)				QOL affected in total, n (%)
		Non (0)	Mild (1-4)	Moderate (5-7)	Severe (8-10)	
Filling symptoms						
1 Frequency	207 (52.1)	258 (65.0)	36 (9.1)	58 (14.6)	45 (11.3)	139 (35.0)
2 Nocturia	333 (83.9)	251 (63.2)	37 (9.3)	54 (13.6)	55 (13.9)	146 (36.8)
3 Urgency	186 (46.9)	292 (73.6)	34 (8.6)	47 (11.8)	24 (6.0)	105 (26.4)
Voiding symptoms						
4 Bladder pain	126 (31.7)	304 (76.6)	25 (6.3)	38 (9.6)	30 (7.6)	93 (23.4)
5 Hesitancy	45 (11.3)	371 (93.5)	8 (2.0)	14 (3.5)	4 (1.0)	26 (6.5)
6 Straining	17 (4.3)	385 (97.0)	6 (1.5)	3 (0.8)	3 (0.8)	12 (3.0)
7 Stop and start during micturition	108 (27.2)	350 (88.2)	14 (3.5)	25 (6.3)	8 (2.0)	47 (11.8)
Incontinence symptoms						
8 Urge incontinence	41 (10.3)	362 (91.2)	12 (3.0)	16 (4.0)	7 (1.8)	35 (8.8)
9 Mixed incontinence	164 (41.3)	301 (75.8)	30 (7.6)	40 (10.1)	26 (6.5)	96 (24.2)
10 Stress incontinence	179 (45.1)	297 (74.8)	30 (7.6)	42 (10.6)	28 (7.1)	100 (25.2)
11 Unpredictable incontinence	23 (5.8)	375 (94.5)	11 (2.8)	5 (1.3)	6 (1.5)	22 (5.5)
12 Enuresis	15 (3.8)	384 (96.7)	3 (0.8)	8 (2.0)	2 (0.5)	13 (3.3)

**Figure 1:**
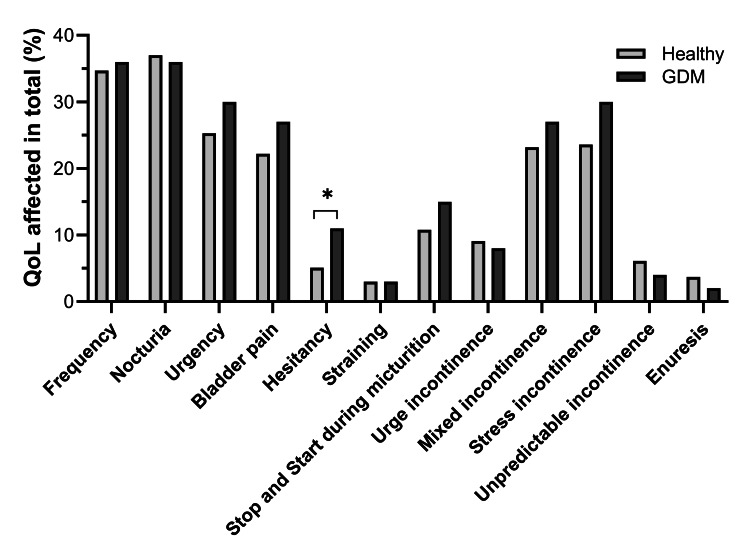
Comparison of QOL impairment between healthy pregnant women and those with diabetes. GDM: gestational diabetes mellitus; QOL: quality of life. Chi-square test; *p < 0.05.

The bother scores, which reflect the perceived impact of symptoms on daily life, are also presented in Table 2. Nocturia was associated with the highest bother score (36.8%). Notably, the proportion of women with GDM who reported a negative impact on QOL due to hesitancy was significantly higher than those without GDM (11.0% vs. 5.1%; p = 0.038) (Figure 1).

Multivariate logistic regression analysis was performed to determine the independent predictors of bladder filling, voiding, and urinary incontinence symptoms in pregnant women. As shown in Table 3, advancing gestational age was significantly associated with an increased likelihood of bladder filling symptoms. Specifically, each additional week of gestation was associated with a 9% increase in odds (OR = 1.09, 95% CI: 1.04-1.15; p < 0.001). Although women with a gravidity of four had lower odds of reporting bladder filling symptoms compared to those with a gravidity of one (OR = 0.28, 95% CI: 0.07-1.10), this association did not reach statistical significance (p = 0.068). Similarly, urinary incontinence symptoms were significantly associated with gestational age. For each additional week, the odds of reporting incontinence increased by 2% (OR = 1.02, 95% CI: 1.00-1.05; p = 0.044). While a higher BMI was also associated with increased odds of urinary incontinence, this finding was not statistically significant (OR = 1.04, 95% CI: 1.00-1.10; p = 0.078). Finally, as presented in Table 3, none of the demographic or clinical variables showed a significant association with voiding symptoms (p > 0.05).

**Table 3:** The likelihood of all symptoms in our study by a range of demographic factors. aOR: adjusted odds ratio; BMI: body mass index; CI: confidence interval. p < 0.05 is significant.

Factors	aOR	95% CI	p-value
Storage symptoms			
Age	1.02	0.94-1.10	0.677
BMI	1.04	0.94-1.16	0.391
Gestation	1.09	1.04-1.15	<0.001
Second gravidity	1.00	0.34-2.94	0.997
Third gravidity	0.74	0.20-2.78	0.657
Fourth gravidity	0.28	0.07-1.10	0.068
Diabetes	0.67	0.26-1.74	0.409
Voiding symptoms			
Age	0.98	0.94-1.02	0.278
BMI	1.00	0.96-1.05	0.954
Gestation	1.02	1.00-1.04	0.110
Second gravidity	0.94	0.57-1.54	0.798
Third gravidity	1.63	0.85-3.10	0.138
Fourth gravidity	1.54	0.74-3.21	0.247
Diabetes	1.17	0.72-1.89	0.538
Incontinence symptoms			
Age	1.02	0.98-1.06	0.448
BMI	1.04	1.00-1.10	0.078
Gestation	1.02	1.00-1.05	0.044
Second gravidity	0.99	0.60-1.65	0.972
Third gravidity	1.33	0.70-2.54	0.387
Fourth gravidity	1.37	0.65-2.86	0.405
Diabetes	1.13	0.69-1.86	0.614

## Discussion

LUTS are commonly observed in women and have a significant adverse impact on their QOL [1], with pregnancy-related changes contributing to their development [16]. Several studies have investigated urinary symptoms in pregnant women from countries such as Palestine, China, Taiwan, and Turkey [17], but no studies have been conducted in Iran focusing on this issue among pregnant women. This study used the validated ICIQ-FLUTS questionnaire to assess LUTS in pregnant women. Previous research has confirmed the reliability and validity of the Persian version of this questionnaire, making it a valuable tool for both epidemiological studies and clinical applications in Iran [13]. Furthermore, this study is the first to explore LUTS and predictors of LUTS, including bladder filling, voiding, and incontinence symptoms, among pregnant women in Yazd, offering unique insights into the prevalence and impact of these symptoms in this specific region.

The results of our study show that LUTS are highly prevalent among pregnant women in Yazd, with nocturia being the most common symptom, reported by 83.87% of participants. Nocturia, defined as waking up to urinate one or more times during the night, is highly prevalent among pregnant populations globally. For instance, studies from various countries report nocturia in 60-80% of pregnant women [16,18]. The high prevalence of nocturia in this study may be attributed to the physiological changes of pregnancy, such as increased renal blood flow and the growing pressure on the bladder from the expanding uterus. The symptom of nocturia, although common, has a significant impact on the well-being of pregnant women, especially in terms of disrupted sleep and decreased overall QOL [19]. This is evident from the bother scores in this study, where nocturia was reported as the most bothersome symptom. The prevalence of nocturia and its associated impact on QOL emphasize the need for healthcare providers to assess and address these symptoms during prenatal visits routinely.

Urinary incontinence, particularly SUI and mixed urinary incontinence, was also highly prevalent in our study, with SUI found in 45.1% of participants. These results are consistent with findings from other studies, which report high rates of incontinence during pregnancy, ranging from 30% to 50% [20,21]. Pregnancy-induced changes in pelvic floor muscle function, hormonal fluctuations, and increased intra-abdominal pressure from the growing uterus likely contribute to the high rates of urinary incontinence observed in this study [22].

The analysis in this study identified advancing gestational age as a significant predictor of both bladder filling and urinary incontinence symptoms. Specifically, each additional week of gestation was associated with a 9% increase in the odds of bladder filling symptoms and a 2% increase in the odds of incontinence symptoms. These findings are consistent with previous literature indicating that as pregnancy progresses, the enlarging uterus and associated pressure on the bladder exacerbate LUTS [23]. Although the association between higher BMI and urinary incontinence symptoms did not reach statistical significance in our study (p = 0.078), the observed trend toward increased odds (OR = 1.04, 95% CI: 1.00-1.10) suggests a potential link that warrants further investigation. Increased BMI is known to elevate intra-abdominal pressure, which can exert additional stress on the pelvic floor muscles and the urethral sphincter, potentially compromising continence mechanisms. Previous studies have consistently reported obesity as an independent risk factor for urinary incontinence, particularly stress incontinence, in both pregnant and non-pregnant populations [24]. Therefore, although our findings did not achieve statistical significance, likely due to sample size or confounding factors, the biological plausibility and consistency with existing literature highlight the importance of monitoring and managing weight as part of incontinence prevention strategies during pregnancy.

Interestingly, our findings indicate that the prevalence of voiding symptoms, such as hesitancy (11.33%), straining (4.28%), and interrupted stream (27.20%), is lower compared to storage symptoms. This suggests that while storage symptoms, particularly nocturia and frequency, are more common in pregnancy, voiding symptoms persist and contribute to the overall burden of LUTS. Although previous studies reported a lower prevalence of voiding symptoms [3,25], the higher prevalence observed in our study may be attributed to differences in study populations and data collection tools. Furthermore, the fact that 31.7% of participants reported bladder pain highlights the multifactorial nature of LUTS during pregnancy [5]. Although previous studies have primarily focused on urinary incontinence [3], our study emphasizes the importance of considering other voiding symptoms, such as hesitancy and bladder pain, which are often overlooked in clinical practice. Despite being less common, these symptoms can significantly affect a pregnant woman’s comfort and QOL.

The study’s findings on bother scores are significant. Nocturia was the most bothersome symptom for pregnant women, followed by urgency and stress incontinence. These results align with previous research that has shown that urinary symptoms, particularly nocturia and incontinence, can significantly impact a woman’s physical and emotional well-being during pregnancy [26,27]. The significant impact of these symptoms on QOL highlights the need for healthcare providers to be vigilant in identifying and managing LUTS in pregnant women to improve their QOL. The significantly higher prevalence of QOL impairment due to hesitancy in women with GDM is noteworthy and may suggest an underlying neurogenic component or altered bladder function in this subgroup. Prior studies have also shown that diabetes may affect bladder innervation and urodynamic function [28], which could partially explain this association.

Management of LUTS in pregnant women should be tailored to minimize discomfort without compromising fetal health. Conservative strategies, such as pelvic floor exercises, bladder training, and fluid management, are often recommended to alleviate symptoms such as nocturia, urinary incontinence, and frequency [29]. In more severe cases, medical or surgical treatments may be considered. Given the high prevalence and substantial impact of LUTS, routine screening and early identification of bothersome symptoms should be integrated into prenatal care to ensure timely intervention and improve maternal QOL [30].

Strengths and limitations

One of the strengths of this study is the relatively large sample size and the comprehensive assessment of LUTS using a validated and culturally adapted questionnaire (ICIQ-FLUTS). However, the cross-sectional nature of the study limits the ability to draw causal inferences. Additionally, the reliance on self-reported data may have introduced recall bias. Although gestational age was analyzed as a continuous variable, we did not report LUTS prevalence by trimester, which may have limited the clinical interpretability of symptom patterns across different stages of pregnancy. Future longitudinal studies are warranted to track the progression and resolution of symptoms across trimesters and into the postpartum period. Finally, the absence of objective assessments (e.g., cough stress test) is another limitation that should be considered in future studies.

## Conclusions

Bladder filling and urinary incontinence symptoms are highly prevalent among pregnant women and are significantly associated with gestational age. Although a higher BMI showed a trend toward increased incontinence, this association was not statistically significant. The findings indicate a high overall prevalence of LUTS among pregnant women in Yazd, with storage symptoms being more common than incontinence and voiding symptoms. Despite their frequency, these symptoms are often overlooked during antenatal care, underscoring the need for greater clinical awareness and early intervention strategies during pregnancy.
